# Clinical Performance of Self-Adhesive vs. Conventional Flowable Resin Composite Restorations in Posterior Teeth: A Systematic Review and Meta-Analysis of Randomized Trials

**DOI:** 10.3390/jcm14165862

**Published:** 2025-08-19

**Authors:** Samille Biasi Miranda, Caroline de Farias Charamba Leal, Giovana Lordsleem de Mendonça, Renally Bezerra Wanderley e Lima, Ana Karina Maciel de Andrade, Rodrigo Barros Esteves Lins, Marcos Antonio Japiassú Resende Montes

**Affiliations:** 1 Department of Dental Materials, Faculty of Dentistry, University of Pernambuco, Recife 50100-130, PE, Brazil; samille.biasi@upe.br (S.B.M.); caroline.charamba@upe.br (C.d.F.C.L.); giovana.lordsleem@upe.br (G.L.d.M.); 2 Department of Restorative Dentistry, Federal University of Paraíba, João Pessoa 58051-900, PB, Brazil; renallywanderley@gmail.com (R.B.W.e.L.); kamandrade@hotmail.com (A.K.M.d.A.); 3 Department of Restorative Dentistry, School of Dentistry, Federal University of Alagoas, Maceió 57072-900, AL, Brazil; rodrigo.lins@foufal.ufal.br (R.B.E.L.)

**Keywords:** self-adhesive flowable composite, dental restoration, direct restoration, resin composite, systematic review

## Abstract

**Background/Objectives**: Self-adhesive flowable resins (SAFR) entered the market, eliminating the adhesive system application due to their self-adhesive technology. Guided by the PICO framework (Population, Intervention, Comparison, Outcome), the aim was to conduct a systematic review of clinical studies to compare the clinical performance of Self Adhesive Flowable Resin (SAFRs) with conventional flowable resins used for direct restorations. **Methods**: The protocol of this systematic review was registered in the International Prospective Register of Systematic Reviews (CRD42023394297) and followed the Preferred Reporting Items for Systematic Reviews and Meta-Analyses guideline. Five databases (PubMed, Embase, Web of Science, Scopus, and Cochrane Library) were searched from inception to July 2025. Nine randomized clinical trials were included, totaling 493 restorations in 232 patients. Clinical performance was assessed using USPHS or FDI criteria, with follow-up periods ranging from 6 months to 5 years. Data were pooled using a random-effects meta-analysis to calculate risk differences (RD) and 95% confidence intervals (CI) for marginal adaptation, retention, marginal staining, post-operative sensitivity, color stability, surface roughness, secondary caries, and anatomical form. **Results**: Meta-analysis showed no significant differences between SAFRs and CFRCs for in terms of: marginal adaptation (RD = 0.01; 95% CI: −0.02 to 0.04; *p* = 0.53; I^2^ = 0%), retention (RD = 0.00; 95% CI: −0.02 to 0.03; *p* = 0.81; I^2^ = 0%), marginal staining (RD = 0.01; 95% CI: −0.01 to 0.02; *p* = 0.51; I^2^ = 0%), and post-operative sensitivity (RD = −0.01; 95% CI: −0.03 to 0.02; *p* = 0.62; I^2^ = 0%). The certainty of the evidence for all outcomes was rated as moderate to high according to the GRADE assessment. **Conclusions**: SAFR restorations demonstrated comparable clinical performance to conventional resins; however, heterogeneity in follow-up duration and the scarcity of long-term data (>5 years) warrant caution.

## 1. Introduction

Composite resins stand as the primary choice among clinicians for restorative materials. As a result, manufacturers persistently work towards enhancing the aesthetic and physical-mechanical properties of these materials. They function by replacing lost dental tissue due to caries or fractures, ensuring aesthetics and functionality for patients [1,2]. The restorative protocol for conventional composite resins involves acid conditioning and adhesive application to prepare the dental substrate. These additional steps make the technique more susceptible to errors and demand more clinical time [3]. Motivated by this, self-adhesive flowable resins (SAFR) entered the market, eliminating the adhesive system application due to their self-adhesive technology, a step prone to errors [4]

The self-adhesiveness of this new class of composite resin is conferred by the addition of the glycerol-phosphate molecule, which conditions the dental structure while also having an affinity for dental element calcium and methacrylate groups that copolymerize with other methacrylate monomers [5]. However, it is important to note that tissue demineralization is partial, meaning there is no complete removal of the smear layer, incorporating it into the adhesive interface, unlike the usually applied technique of total acid conditioning, where the smear layer is entirely removed [6].

SAFRs are indicated for direct restoration repair [7], direct restorative material [8], and as pit and fissure sealants [9]. However, in vitro studies indicate that SAFRs exhibit inferior marginal sealing ability and dentin bond strength values compared to other adhesive systems and conventional flowable resins [3,5]. These findings may be due to the finer resinous tags formed, the presence of the smear layer, and the high viscosity of SAFRs [4,10,11]. In contrast, other in vitro studies have demonstrated that SAFRs exhibit superior marginal sealing compared to all-in-one adhesive systems in class II cavities, and similar marginal adaptation to conventional flowable in class I cavities [12,13]. These results have been attributed to the lower hygroscopic expansion and polymerization shrinkage of SAFRs [13]. Clinical trials have revealed the potential use of SAFRs as restorative materials in Class I cavities, demonstrating notable clinical effectiveness regarding retention, secondary caries, and marginal discoloration [8]. However, there is a chance of suboptimal performance in terms of marginal adaptation and polishing [14]. In Non-Carious Cervical Lesions, the restorations exhibit a high failure rate [15]. Based on the available evidence from clinical and laboratory research, it is currently impossible to determine whether SAFRs possess favorable properties and clinical efficacy, thus casting doubt on their suitability for clinical use. Therefore, it is necessary to compile current scientific evidence on SAFR’s performance.

To the best of our knowledge, no previous meta-analysis has exclusively synthesized clinical evidence from randomized controlled trials evaluating self-adhesive flowable resin composites for direct restorations in posterior teeth. Previous reviews have primarily addressed in vitro outcomes such as bond strength, microleakage, and marginal adaptation [3], which cannot fully predict long-term clinical behavior, making the present review the first to focus on clinically applicable analysis of SAFR performance, overcoming the methodological heterogeneity and indirectness of previous reviews. However, the current literature is limited by a scarcity of long-term follow-up data, with most available clinical trials reporting outcomes within 2 years and very few extending to 5 years. This gap hinders a comprehensive understanding of the true longevity and failure patterns of these materials over extended periods.

There is a necessity to conduct an evidence synthesis study that examines data from multiple primary clinical studies. Such a study enables the generation of robust and dependable scientific evidence concerning the clinical suitability of this new material. This effort aims to offer stronger scientific evidence regarding the clinical performance of this new restorative material and to affirm or contest it is for use. Thus, the objective of this systematic review was to compare the clinical performance of self-adhesive flowable resins (SAFRs) and conventional flowable composites in direct restorations

## 2. Materials and Methods

### 2.1. Protocol and Registration

This systematic review was conducted following the guidelines of the PRISMA (Preferred Reporting Items for Systematic Reviews and Meta-Analyses) guidelines (Supplementary Material Table S1) [16] and was structured as follows: (1) identification of the guiding question, (2) gathering of relevant studies, (3) determination of inclusion and exclusion criteria, (4) data extraction, and (5) synthesis of results [17]. Prior to commencement, the methodology of this study was registered in PROSPERO (International Prospective Register of Systematic Reviews) with the protocol number CRD42023394297.

### 2.2. Eligibility Criteria

The guiding question for this review was “Do SAFRs have clinical performance comparable to conventional flowable resins as direct restorative materials”? The Population/Problem, Intervention/Exposure, Comparison, and Outcome of the study were guided by the PICO strategy. The Population (P) consisted of patients with restorations in posterior teeth. The Intervention (I) analyzed was direct restoration with SAFRs, and the Comparator (C) was conventional flowable resin. The Outcomes (O) evaluated were marginal adaptation, retention, marginal staining, post-operative sensitivity, color stability, surface roughness, smoothness, secondary caries, and anatomical form.

Inclusion criteria for the studies were: (1) randomized clinical trials evaluating the clinical performance of SAFRs as direct restorative materials; (2) a follow-up period longer than 5 months was established to ensure the inclusion of studies with adequate time to detect early clinical failures while still retaining a sufficient number of trials for synthesis, shorter follow-up times (≤ 6 months) may underestimate early failure rates, whereas adopting a ≥ 1-year cutoff would have excluded a substantial portion of available evidence. Exclusion criteria were: (1) studies evaluating SAFRs only regarding post-operative sensitivity were excluded, as this isolated parameter does not comprehensively reflect the clinical performance of a restorative material; only studies reporting a broader range of functional, biological, and esthetic outcomes were considered; (2) studies assessing the ability of SAFRs as sealing agents were also excluded because the primary objective was to evaluate their performance as restorative materials subjected to occlusal and proximal load, which differ in mechanical and esthetic demands from preventive sealant applications; (3) studies that included experimental SAFRs; (4) non-inferiority trials were excluded because their statistical design and interpretation aim to confirm that a treatment is not worse than a comparator by a pre-specified margin, rather than to directly compare and quantify differences in performance, since this review adopted a comparative effectiveness approach, only trials designed to detect superiority or equivalence in clinical outcomes were included; (5) unpublished information in the scientific literature; (6) studies with unavailable full text.

### 2.3. Information Sources and Search Strategy

PubMed, Embase, Web of Science, Scopus, and Cochrane were accessed on 2 July 2025, using a search strategy that did not limit a chronological period to find clinical studies evaluating the use of self-adhesive flowable composites as direct restorative materials. The following Medical Subject Headings (MESH) or text words were used: self-adhesive composite, self-adhering composite, self-adherent composite, permanent dental restorations, permanent dental restoration, permanent dental filling, flowable hybrid composite, flowable composite, and flowline. The following search was performed across all databases: [(“Self adhering composite” OR “self adhesive composite” OR “self-adherent composite”) AND (“restorations, permanent dental” OR “restoration permanent dental” OR “dental permanent filling”)] AND [“flowable hybrid composite” OR “hybrid composite flowable” OR “flowline”] (Supplementary Material Table S2).

### 2.4. Selection Process

Studies were saved and systematically organized using the online program Rayyan (Qatar Computing Research Institute, Ar-Rayyan, Qatar) [18]. Duplicates were first removed, and then titles and abstracts were read to determine if the studies met the predefined criteria. The selection process was conducted independently by two authors (S.B.M., and C.F.C.L), previously calibrated, and discrepancies were discussed with a third author (G.L.d.M). The calibration process involved the two independent reviewers initially reading and assessing a set of 10 articles together to ensure alignment in their understanding and application of the selection criteria. Through this collaborative review, they established a consistent approach to article selection based on the predetermined criteria. Subsequently, any disparities or discrepancies encountered during the independent selection process were addressed through discussion and consensus with the third author. Eligible articles were read in full, and their data were extracted.

### 2.5. Data Collection Process

Three authors, previously calibrated, performed data extraction using a guiding table covering the main methodological characteristics of the studies. Key data included author, publication year, self-adhesive composite used, volunteers, number of restored teeth, type of restored tooth, cavity type, study design, follow-up period, analysis criteria, and conclusion.

### 2.6. Study Risk of Bias Assessment

Included studies underwent a risk of bias analysis by two independent reviewers, previously calibrated. The Cochrane Risk of Bias for Randomized Trials version 2 (RoB 2) tool was used, structured into domains analyzing bias arising from the randomization process, deviations from intended interventions, missing outcome data, measurement of outcome, and selection of reported results [19]. For each domain, there are signaling questions that are designed to offer a systematic method for extracting information pertinent to assessing the risk of bias and offer as answers: yes, probably yes, probably no, no, no information [20]. After responding to the signaling questions, the next step is to reach a risk-of-bias judgment, as follows: Low risk of bias, some concerns, or high risk of bias [19]. The RoB 2 tool incorporates algorithms that link responses to signaling questions to a suggested risk-of-bias assessment for each domain [19]. In case of disagreement between the two assessors, a third assessor was consulted to reach a consensus.

### 2.7. Effect Measures and Synthesis Methods

Meta-analysis was performed using a random-effects model. Review Manager version 5.4 (Cochrane collaboration) was used to calculate the risk difference with a 95% confidence interval. For this analysis, data were dichotomized. A random-effects model was used because clinical and methodological variability was expected among the included trials, particularly in follow-up duration, patient populations, and evaluation criteria (USPHS vs. FDI). Statistical heterogeneity was assessed using the I^2^ statistic, with values below 50% considered low, between 50% and 75% moderate, and above 75% high. All pooled analyses in this review presented I^2^ < 50%, indicating low heterogeneity. The acceptable restorations were those that received the Alpha and Bravo scores. The unacceptable restorations were those that received the Charlie score in at least one clinical parameter of the USPHS (marginal adaptation, retention, sensitivity, color stability, smoothness, secondary caries, and anatomical form). The clinical parameters evaluated by the FDI criteria that coincide with the parameters assessed by the USPHS were also included. In this case, scores 4 and 5 were considered, comparable to the Charlie score, and these data were used in the meta-analysis. Table 1 outlines the USPHS assessment criteria, and Table 2 outlines FDI assessment criteria.

### 2.8. Certainty Assessment

Certainty of evidence was assessed for each outcome using the Grading of Recommendations: Assessment, Development, and Evaluation (GRADE) tool (http://www.gradeworkinggroup.org/), accessed on 17 July 2025. This tool categorizes the study design and addresses risk of bias, imprecision, inconsistency, indirectness of evidence, and publication bias to possibly classify the quality of evidence. Each of these items is assessed as “no limitation”, “serious limitations”, and “very serious limitations” to categorize the evidence quality as high, moderate, low, or very low. Lower quality suggests that the estimate is far from the actual effect.

## 3. Results

### 3.1. Study Selection

**Figure 1:**
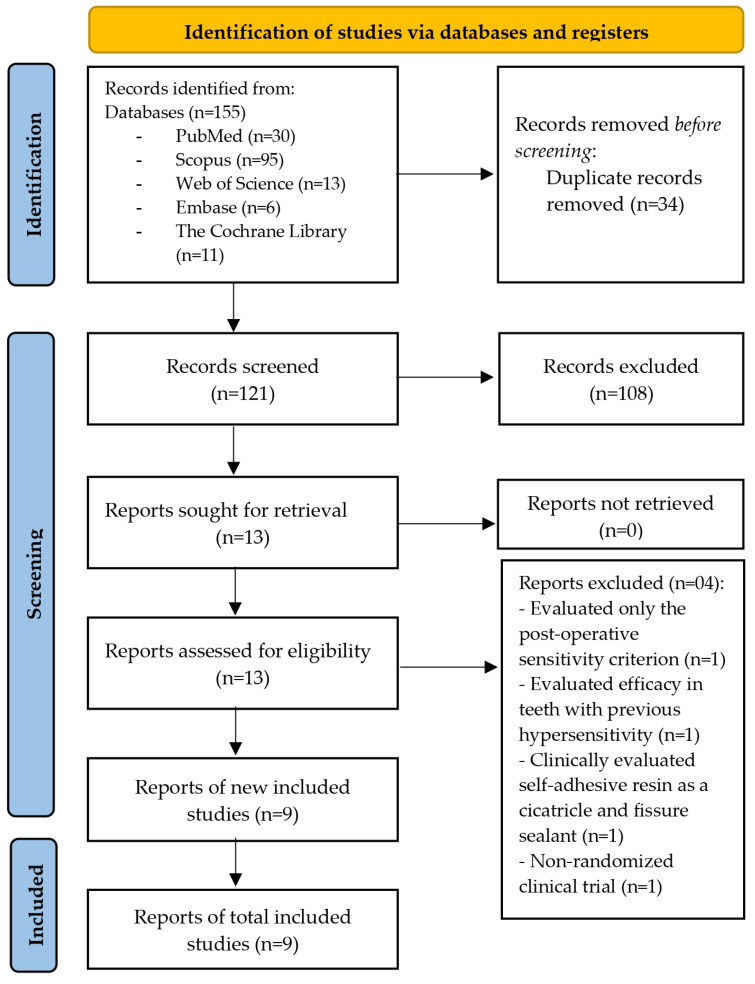
Flow diagram of literature search and selection criteria.

A total of 155 studies were extracted from the databases in the search conducted in July 2025. After removing duplicates, 121 studies remained, and their titles and abstracts were analyzed according to the predefined inclusion and exclusion criteria. Full article reading was conducted on 13 studies considered potentially eligible, of which 9 met the selection criteria (Figure 1). One study was excluded for assessing only post-operative sensitivity criteria [20], another for evaluating effectiveness in teeth with previous hypersensitivity [21], for clinically evaluating self-adhesive resin as a pit and fissure sealant [9], and the last due to being a non-randomized clinical study [15].

### 3.2. Study Characteristics

The characteristics of the studies are observed in Table 3.

The included articles were published between 2015 and 2022. The number of participants varied between 18 and 37. The ages of the study volunteers ranged from 4 to 79 years, with follow-up periods from 6 months to 5 years [8,14,22,23,24,25,26,27,28] The countries where the studies were conducted were Saudi Arabia [24], Poland [14], Turkey [22,26,27], Lebanon [23], Egypt [25,28] and India [8].

The total number of treated patients and restored teeth was 232 and 493, respectively. In seven studies, class I cavities were performed [8,14,23,25,26,27,28], and in two, class V cavities were performed [22,24], one non-carious cervical lesion [22] and another cervical carious lesion [24]. The restored dental elements included molars, incisors, canines, premolars, and deciduous molars. Of the selected studies, only Çelik, Aka, Yilmaz [23] and Oz et al. [27] used the FDI criteria, and the others used the USPHS. The SAFRs used were Vertise Flow [14,23,24,25,27,28], Fusion Liquid Dentin [8,22,24]. The conventional flowable resins used were Premise Flowable [14,22,24], Tetric N Flow [8,24], Filtek z350 Xt [27,28], Filtek z250 [26], LuxaFlow [27]. Almost all studies reported using absolute isolation, except for Maj et al. [14] and Oz et al. [27]. Çelik, Aka, and Yilmaz [22] performed relative isolation. One study also evaluated the influence of using absolute isolation or cotton rolls [23].

For the meta-analysis, the data for the proportion of restorations considered with worse scores (Charlie) were extracted from the studies that applied the United States Public Health Service (USPHS) criteria and the data for the restorations with the equivalent worse scores (score 4 and 5) were extracted from studies that applied the World Dental Federation (FDI), in the following criteria: adequate marginal adaptation, retention, sensitivity, marginal pigmentation, color match, surface roughness, polishing, recurrence of caries, and anatomical form (Table 4). Variables not analyzed were filled in the table with “not mentioned”.

**Figure 2:**
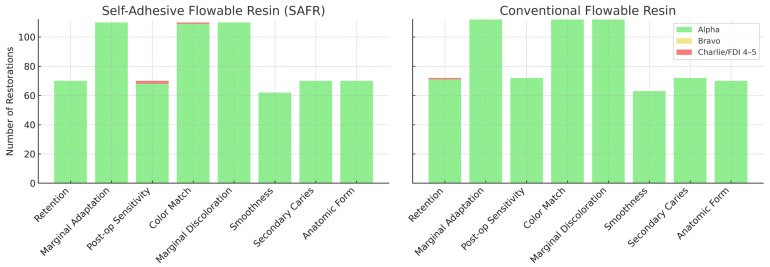
Distribution of clinical scores by resin type for each evaluated outcome. Distribution of clinical scores by resin type for each evaluated outcome. Stacked bar graphs showing the proportion of Alpha (green, clinically successful), Bravo (yellow, clinically acceptable), and Charlie/FDI 4–5 (red, clinically unacceptable) scores for self-adhesive flowable resin (SAFR) and conventional flowable resin restorations across all clinical parameters.

The distribution of clinical scores for each evaluated outcome is presented in Figure 2. The stacked bar graphs enable a visual comparison between self-adhesive flowable resin (SAFR) and conventional flowable resin restorations, complementing the numerical synthesis from the meta-analysis. In both groups, the vast majority of restorations received Alpha scores (clinically successful), followed by a small proportion of Bravo scores (clinically acceptable), and very few Charlie/FDI 4–5 scores (clinically unacceptable), reinforcing the finding of comparable clinical performance between the two materials.

### 3.3. Risk of Bias in Studies

**Figure 3:**
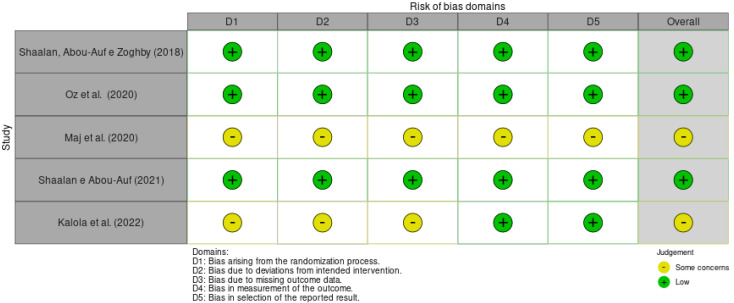
Bias Risk Analysis for the randomized clinical trials [8,14,25,27,28].

The RoB 2 assessment indicated that, although most randomized clinical trials presented a low overall risk of bias, some methodological limitations were identified. Two studies did not describe the randomization process, and four did not report the method of allocation concealment. Incomplete information was also observed regarding the blinding of participants, examiners, and staff in two studies, as well as follow-up losses. Additionally, one study did not clarify whether examiners were aware of the allocation and lacked a pre-specified analysis plan. These potential sources of bias should be considered when interpreting the results (Figure 3).

### 3.4. Results of Syntheses

**Figure 4:**
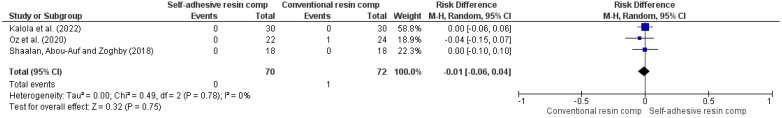
Forest plot of retention (Risk Difference, 95% CI, random-effects model) [8,25,27].

**Figure 5:**
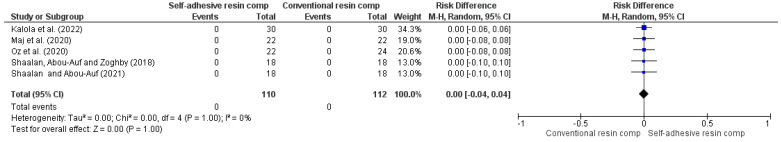
Forest plot of marginal adaptation (Risk Difference, 95% CI, random-effects model) [8,14,25,27,28].

**Figure 6:**
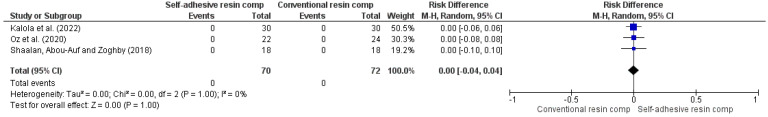
Forest plot of post-operative sensitivity (Risk Difference, 95% CI, random-effects model) [8,25,27].

**Figure 7:**
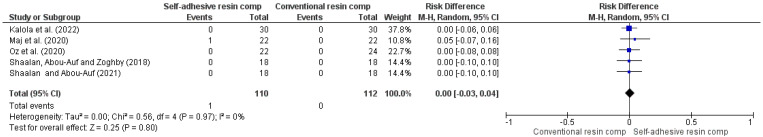
Forest plot of staining (Risk Difference, 95% CI, random-effects model) [8,14,25,27,28].

**Figure 8:**
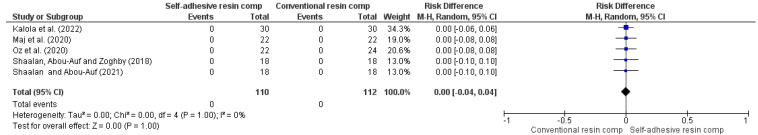
Forest plot of marginal discoloration (Risk Difference, 95% CI, random-effects model) [8,14,25,27,28].

**Figure 9:**
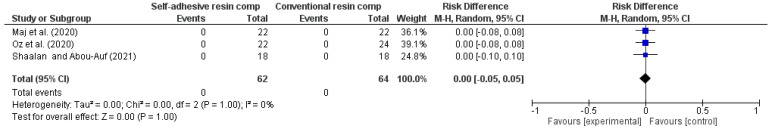
Forest plot of smoothness (Risk Difference, 95% CI, random-effects model) [14,27,28].

**Figure 10:**
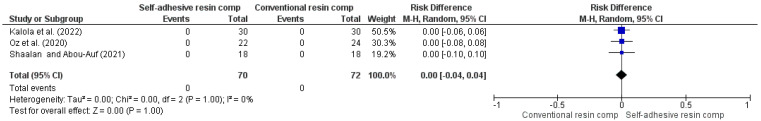
Forest plot of dental caries (Risk Difference, 95% CI, random-effects model) [8,27,28].

**Figure 11:**
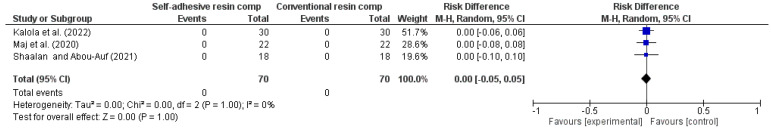
Forest plot of anatomic form (Risk Difference, 95% CI, random-effects model) [8,14,28].

The meta-analysis was conducted exclusively for Class I and II cavities because only one study evaluated carious lesions in Class V cavities, and another study assessed non-carious cervical lesions. As a result, it was impossible to include these studies in the same meta-analysis. The meta-analysis included the five studies selected in the systematic review [8,14,25,27,28]. No significant differences were observed between conventional resin composites and SAFRs for the outcomes studied (*p* > 0.05) (Figure 4, Figure 5, Figure 6, Figure 7, Figure 8, Figure 9, Figure 10 and Figure 11).

Descriptive analysis of the data not included in the meta-analysis [8,14,22,26] revealed a serious report on the lack of retention (27 out of 40 restorations) of SAFRs in non-carious cervical lesions [22]. In Class V cavities (carious cervical lesions), no statistical difference was found with conventional resin used as a control [24]. In deciduous teeth, Serin et al. [26] reported no restoration failures in the evaluated clinical parameters (retention, post-operative sensitivity, marginal staining, and anatomical form). The number of restorations with an alpha score (success) decreased over time; however, no performance difference was found between SAFR and conventional flowable resin after 2 years of evaluation.

### 3.5. Certainty of Evidence

In Table 5, it is shown that the certainty of the evidence of marginal adaptation, retention, post-operative sensitivity, staining risk, smoothness, secondary caries, anatomic form, and marginal discoloration was rated as high for all follow-up periods. Table 6 provides a summary of findings (SAFRs vs. CFR in posterior restorations).

Table 6 presents the Summary of Findings for this systematic review, compiling the results for the evaluated clinical outcomes (retention, marginal adaptation, marginal discoloration, post-operative sensitivity, anatomic form, secondary caries, surface smoothness, and color match). For each outcome, the table displays the risk ratio (RR), 95% confidence interval (95% CI), number of restorations assessed in each group (SAFR/CFR), *p*-value, and the certainty of the evidence according to the GRADE approach. Across all parameters, no statistically or clinically significant differences were observed between SAFRs and CFRs, and the certainty of the evidence was rated as high, indicating robust findings and a low likelihood that further research would substantially change these conclusions.

## 4. Discussion

This systematic review and meta-analysis of clinical studies aimed to assess the clinical performance of SAFRs as direct restorative materials. The meta-analysis results revealed that SAFRs demonstrate clinically comparable results to conventional composite resins for the outcomes studied (anatomical form, secondary caries, marginal adaptation, color match, smoothness, retention, sensibility, and marginal staining). SAFRs offer practical advantages over conventional flowable resins, particularly a simplified clinical protocol by eliminating the need for separate etching and adhesive application, which reduces chair time and decreases the risk of procedural errors such as over-etching or inadequate moisture control [29]. These benefits can be especially valuable in pediatric or uncooperative patients and in high-volume clinical settings. However, evidence from NCCL studies indicates that these advantages may be offset by lower retention rates in non-retentive preparations [22], likely due to limited micromechanical interlocking and potential hydrolytic degradation of the adhesive interface. Clinicians should therefore exercise caution when considering SAFRs for NCCLs until more robust long-term data become available.

The findings of this systematic review and meta-analysis, supported by high-certainty evidence, indicate that SAFRs are non-inferior but not superior to conventional flowable resins in posterior Class I restorations. This suggests that clinicians can select SAFRs in situations where simplified application and reduced chair time are priorities, without compromising restoration longevity or quality [8,14,25,27,28,29]. However, for non-retentive preparations such as Class V cavities and NCCLs—where micromechanical retention is minimal—current evidence indicates a higher risk of early failure for SAFRs [22], and conventional systems may remain the preferred choice until further long-term data are available. Therefore, the choice between SAFRs and conventional flowable resins should be individualized, balancing material cost, procedural efficiency, and cavity configuration.

Most include studies of this systematic review and meta-analysis utilized Vertise Flow self-adhesive resin (n = 5) [14,24,25,27,28], followed by Fusion Liquid Dentin (n = 4) [8] (Table 4). Vertise Flow resin bonds to the tooth through the affinity between the Glycerol Phosphate Dimethacrylate (GPDM) monomer and calcium ions in the tooth, as well as through micromechanical bonding between polymerized monomers and collagen fibers in dentin [30]. Fusion Liquid Dentin resin contains the negatively charged 4-Methacryloxyethyl trimellitate anhydride (4-META) monomer, which links to tooth ions, incorporating into dentin and improving adhesion and sealing [31]. The clinical performance of SAFRs was evaluated in Class I (n = 5) [8,14,25,27,28], Class V (n = 1) [24], and Non-Carious Cervical Lesion (NCCL) (n = 1) [22] cavities. Class V cavities and NCCL, compared to Class I, lack micromechanical retention in their cavity configuration, favoring analyses related to adhesion [15].

The meta-analysis and GRADE assessment showed no statistically or clinically significant differences between SAFRs and conventional flowable resins for any evaluated outcome, with all comparisons rated as high-certainty evidence. These results support the interpretation that SAFRs are non-inferior to conventional resins in terms of clinical performance for posterior Class I restorations. However, the absence of superiority indicates that their use should not be justified on the basis of enhanced longevity or clinical outcomes, but rather on potential advantages such as simplified application and reduced operative time [8,14,24,25,26,27,28].

The most commonly used method for clinical evaluation of resins in the reviewed studies was USPHS, an event that proves to be a trend in clinical studies [26]. This criterion encompasses biological, aesthetic, and functional aspects of the investigated restorative materials through randomized clinical trials [32]. However, another criterion, known as the FDI criteria, is also utilized to monitor the longevity of restorative procedures in clinical trials. A study emphasizes that the variety of scores (1–5) enables a higher potential to detect differences in restorations, thereby enhancing the quality of the evaluation conducted [33]. For the meta-analysis, restorations that obtained the Charlie score (USPHS) or scores 4 and 5 (FDI criteria) in the assessed parameters (anatomical form, secondary caries, marginal adaptation, color match, smoothness, retention, sensibility, and marginal staining) were counted. This score represents the category that considers the restorative procedure as unacceptable or unsuccessful [26,33]. Surface roughness was excluded from the meta-analysis due to the lack of comparative studies, as the analyses were conducted separately according to the type of dental cavity. Therefore, future clinical studies evaluating surface roughness are required.

Retention of SAFRs was comparable with conventional flowable resins in the meta-analysis of this study when compared in posterior teeth (Figure 4). For a restoration’s performance to be considered successful, it needs to have longevity, thus confirming retention as one of the most important criteria [24]. This favorable performance may be related to the type of cavity that tends to be more retentive (Class I and II) [28]. Despite these results, in the study by Çelic, Aka, and Yılmaz [22], a failure rate of 67.5% was found in SAFR retention. This event may be attributed to the hydrolytic degradation of the resin-dentin interface and lower conditioning capacity of SAFR [15,21,22]. More specifically, the poorer performance of Fusion Liquid Dentin may be related to the hydrolytic instability of 4-META. Additionally, this study evaluated the performance of SAFR in NCCL’s cavities that lack micromechanical retention, a factor that may reveal the true behavior of the material regarding adhesiveness and must be the subject of future studies. Alongside retention, marginal adaptation clarifies the sealing ability of restorative materials [22].

The included trials assessed SAFRs in different cavity configurations, predominantly Class I restorations [8,14,25,27,28], but also in Class V cavities [24] and non-carious cervical lesions (NCCLs) [22]. This variability is clinically relevant because Class I preparations are inherently more retentive, potentially masking limitations in adhesive performance, whereas Class V and NCCLs rely heavily on the bonding system due to the lack of mechanical retention. The markedly higher early failure rate reported for SAFRs in NCCLs [22] suggests that outcomes observed in retentive preparations cannot be directly extrapolated to non-retentive lesions. Therefore, future clinical trials should stratify results by cavity type to better inform material selection in different clinical scenarios.

The markedly higher early failure rate reported for SAFRs in NCCLs [22] and the limited data available for Class V restorations [24] highlight the need for targeted research in these lesion types. Non-retentive preparations present a more demanding test of adhesive performance, as retention relies almost entirely on the quality and durability of the resin–tooth interface. Early debonding in such lesions may indicate limitations in the current formulations of SAFRs and underscores the importance of long-term trials that specifically address these clinical scenarios. Understanding the mechanisms and prevalence of early failure in these contexts is crucial for guiding appropriate material selection.

Following the same pattern, the marginal adaptation (Figure 5) of SAFRs was comparable to that of conventional flowable resins. The comparable performance of SAFRs can be attributed to their chemical composition, including the presence of GPDM (for conditioning dentin and enamel), HEMA, amorphous silica, and nano-sized glass. Additionally, as the carboxylic acids of the material are neutralized and the monomers polymerize, they integrate into the dentin surface, enhancing its sealing capacity [24,34]. In regard to post-operative sensitivity, SAFRs also showed comparable results to the control group (Figure 6).

The absence of post-operative sensitivity is related to the absence of acid conditioning and smear layer removal, keeping dentinal tubules sealed [35]. The literature highlights that eliminating the need for separate acid conditioning and adhesive application reduces the risk of excessive conditioning, excessive wetting, or excessive drying, factors that can cause the collapse of collagen fibers [15]. The success of a restorative procedure is also related to color stability over time [34]. Regarding discoloration of the restoration, SAFRs showed comparable performance when analyzing the resin color during the follow-up period of the studies included in the meta-analysis (Figure 7). The greater color change in conventional flowable resin may be related to the larger size of its particles, making it more susceptible to discoloration [24]. This fact may also justify the similar results regarding marginal discoloration (Figure 8).

A roughness surface texture may promote plaque accumulation and increase the risk of secondary caries, with this characteristic being influenced by the composition and size of the fillers present in composite resins. SAFRs exhibit good surface finishing, possibly due to the presence of nano-sized silica and glass [24]. This characteristic justifies the comparable performance of SAFRs to conventional resins in terms of surface smoothness and secondary caries occurrence (Figure 9 and Figure 10). Another factor influencing the recurrence of secondary caries is the interface stress between the resin material and the tooth. The literature mentions that stress between SAFR and the tooth is insufficient to cause restoration separation and subsequent marginal infiltration, likely due to its low modulus of elasticity, thereby reducing the risk of secondary caries formation. Regarding anatomic form, SAFR exhibits similar performance comparable to conventional resin (Figure 11). Azizi et al. [36] found similar results. Fluid resins tend to exhibit good behavior regarding wear due to the presence of small particles and intra-particle spaces that protect the matrix [36]. There are few studies investigating the wear resistance of SAFRs, making it difficult to gain a greater understanding of the compositions and characteristics of these resins.

Regarding the analysis of bias in the studies, most were classified as Low concerns [24,26,27], followed by some concerns [8,14]. Studies with a low risk of bias reduce influences that could alter the results, making the findings more reliable and valid. The quality of evidence produced in the meta-analysis was classified as high, originating from RCTs that mostly presented a not serious risk of bias. A high level of evidence demonstrates robustness in the findings of the study and assists in evidence-based decision-making. However, variation in methodological quality was observed among the studies, with a lack of details regarding allocation concealment and blinding in some cases. Although the overall evidence rating was high according to GRADE, these methodological limitations should be considered when interpreting the results.

The absence of statistically and clinically significant differences between SAFRs and conventional flowable resins across all evaluated parameters, combined with the high-certainty ratings from the GRADE assessment, supports the conclusion of non-inferiority for SAFRs in posterior Class I restorations. Clinically, this means that practitioners may choose between these materials based on factors such as handling characteristics, application time, and patient-specific considerations, rather than expecting a difference in restoration longevity or performance. However, the interpretation of non-inferiority should be tempered by the limited number of studies and relatively short follow-up periods, which may not capture late failures.

Based on the findings of this study, SAFRs showed comparable clinical performance compared to conventional flowable resins in terms of retention, marginal adaptation, post-operative sensitivity, marginal discoloration, anatomic form, secondary caries, smoothness, and color stability. This study had the limitation of including studies with a follow-up time between 6 months to 5 years; differences in the effectiveness of the therapies can only be measured with greater accuracy over longer periods (≥10 years), as many clinical failures of restorations manifest late [37]. Most of the included studies had relatively short follow-up durations (≤2 years), which may not adequately capture long-term clinical performance or failure rates. Additionally, the limited number of published trials and the absence of registered but unpublished studies raise the possibility of publication bias, which could further influence the overall conclusions.

Some studies included in the meta-analysis used the USPHS criterion, whose sensitivity is limited and may not definitively determine whether such a restorative procedure was successful or not [35]. The FDI criteria appear to be more sensitive and accurate than the USPHS criteria in detecting small differences in clinical studies [36]. Another limitation was the scarcity of studies in non-retentive cavities (Class V and NCCLs), as these are more ideal for studying the adhesive properties of a restorative material [22]. The findings of this systematic review and meta-analysis should be evaluated with caution due to the limited number of available clinical studies. We suggest conducting more randomized clinical studies to assess the clinical capability of these new flowable resins introduced to the market as direct restorative materials, particularly with a longer follow-up period, as the number of events tends to be lower in initial assessments.

Although cost-effectiveness was not assessed in the present review, it remains an essential factor for the clinical adoption of new restorative materials. SAFRs may offer indirect cost savings by reducing operative time and the need for multiple application steps, potentially increasing productivity in busy practices. However, these benefits must be weighed against the potentially higher unit price of SAFR materials compared to conventional flowable resins. Without formal economic evaluations, it is not possible to determine whether SAFRs represent a cost-effective alternative in routine practice. Future studies should incorporate health-economic analyses alongside clinical outcomes to better inform decision-making. In addition, only seven randomized clinical trials met the inclusion criteria for the meta-analysis, which reduces the statistical power and the generalizability of the findings. Therefore, the non-significant results should be interpreted with caution, considering the possibility of failing to detect a true difference between the groups due to the limited sample size. Considering the possibility of failing to detect a true difference between the groups due to the limited sample size. Future research should specifically address these clinical contexts, particularly non-retentive preparations such as Class V cavities and NCCLs, through well-designed randomized controlled trials with larger sample sizes, standardized outcome measures, and extended follow-up periods. Such studies are essential to confirm the long-term performance of SAFRs and to guide their appropriate use across different restorative scenarios.

## 5. Conclusions

Within the limitations of this systematic review and meta-analysis, self-adhesive flowable resins demonstrated clinical performance comparable to that of conventional flowable resins across all evaluated parameters, including retention, marginal adaptation, post-operative sensitivity, marginal discoloration, anatomic form, secondary caries, smoothness, and color stability. However, most available studies have short follow-up periods, and evidence from long-term clinical trials is scarce. Further well-designed randomized controlled studies with extended observation periods are necessary to confirm the durability and long-term effectiveness of these materials in clinical practice.

## Figures and Tables

**Table 1:** USPHS criteria.

The USPHS Criteria
Retention Alpha (A): Restoration is present. Delta (D): Restoration is partially or totally missing. Color match Alpha (A): The restoration matches the adjacent tooth tissue in color, shade, or translucency. Bravo (B): There is a slight mismatch in color, shade, or translucency, but within the normal range of the adjacent tooth structure. Charlie (C): There is a slight mismatch in color, shade, or translucency, but outside of the normal range of the adjacent tooth structure.
Marginal discoloration Alpha (A): There is discoloration anywhere along the margin between the restoration and the adjacent tooth structure. Bravo (B): Discoloration is present, but has not penetrated along the margin in a pulpal direction. Charlie (C): Discoloration has penetrated along the margin in a pulpal direction.
Recurrent caries Alpha (A): No caries are present at the margin of the restoration, as evidenced by softness, opacity, or etching at the margin. Bravo (B): There is evidence of caries at the margin of the restoration.
Surface roughness Alpha (A): The restoration surface is as smooth as the surrounding enamel. Bravo (B): The restoration surface is rougher than the surrounding enamel. Charlie (C): Surface pitting is sufficiently coarse to inhibit the continuous movement of an explorer across the surface.
Marginal integrity Alpha (A): There is no visible evidence of a crevice along the margin into which the explorer penetrates. Bravo (B): There is visible evidence of a crevice along the margin into which the explorer penetrates or catches. Charlie (C): The explorer penetrates the crevice, and dentin or the base is exposed. Delta (D): The restoration is mobile, or missing, either in part or total.
Post-operative sensitivity Alpha (A): Normal reaction to cold spray compared with that of no restored teeth. Bravo (B): Increased cold sensitivity. Charlie (C): Spontaneous pain. Delta (D): Nonvital.

**Table 2:** FDI criteria.

The FDI Criteria
Aesthetic properties Surface gloss Score 1: Gloss similar to enamel. Score 2: Slightly opaque; Some isolated pores. Score 3: Opaque surface, but acceptable if covered by saliva; Multiple pores in more than half of the surface. Score 4: Rough surface, where polishing is not sufficient. Score 5: Very rough surface, unacceptable. Staining Score 1: No superficial or marginal staining. Score 2: Minimal staining, easily removable. Score 3: Moderate staining, also present in other teeth, and aesthetically acceptable. Score 4: Unacceptable staining in the restoration, intervention necessary. Score 5: Severe generalized or localized staining, without access for intervention. Color stability or translucency Score 1: Good coloration and translucency compared to neighboring teeth. Score 2: Minimal color and translucency deviation. Score 3: Clear deviation, but without affecting aesthetics. Score 4: Localized clinical deviation that can be corrected by repair. Score 5: Unacceptable, replacement necessary. Anatomic shape Score 1: Ideal shape. Score 2: Shape slightly deviates from normal. Score 3: Shape differs from normal but does not compromise aesthetics. Score 4: Shape is affected and aesthetically unacceptable. Intervention/correction is necessary. Score 5: Unacceptable or lost. Requires replacement.
Functional properties Fractures and retention Score 1: No fractures or cracks. Score 2: Small crack. Score 3: Cracks that do not affect marginal adaptation. Score 4: Chips that damage marginal adaptation or contact point. Score 5: Partial or total loss of the restoration. Marginal adaptation Score 1: Harmonious line without gaps or discoloration. Score 2: Small marginal fracture, removable with polishing. Score 3: Gap less than 150µm, not removable; Several small fractures in enamel and dentin. Score 4: Gap larger than 250 µm or exposed dentin; Chips damage margin; Noticeable fracture in enamel or dentin. Score 5: Large gaps or widespread irregularities. Anatomic shape Score 1: Normal contact point (dental floss or 25 µm metal foil can pass through; Normal contour. Score 2: Slightly too strong contact but without disadvantage (dental floss or 25 µm metal foil can only pass with pressure); Slightly deficient contour. Score 3: Somewhat weak contact, no indication of damage to the tooth, gum, or periodontal structures; 50 µm metal foil can pass; Visibly deficient contour. Score 4: Too weak and possible damage due to food impaction; 100 µm metal foil can pass; Inadequate contour; Repair possible. Score 5: Too weak and/or clear damage due to food impaction and/or pain/gingivitis; Insufficient contour, requires replacement.
Biological properties Post-operative sensitivity Score 1: No hypersensitivity; normal vitality. Score 2: Low hypersensitivity for a short period of time; normal vitality. Score 3: Moderate hypersensitivity; Weak sensitivity that does not require treatment. Score 4: Intense hypersensitivity; Negative sensitivity; intervention necessary, but no replacement. Score 5: Very intense, pulpitis or nonvital. Endodontics is necessary for restoration or replacement. Caries recurrence Score 1: No secondary or primary caries. Score 2: Small and localized; Demineralization. Score 3: Larger areas of lesion without dentin exposure. Score 4: Caries with cavitation. Score 5: Deep secondary caries or exposed dentin, not accessible for repair or restoration

Score 1: Clinically excellent; Score 2: Clinically good; Score 3: Clinically satisfactory; Score 4: Clinically unsatisfactory, but repairable; Score 5: Clinically poor, replacement is necessary.

**Table 3:** Qualitative summary of the selected studies.

Author (s)	Country	Study Design	Patients (n)	Mean Age (Years)	Teeth Restored	Groups	Tooth Type	Cavity Type	Follow-Up (Months)	Criteria	Conclusion
Çelik, Aka and Yilmaz (2015) [22]	Turkey	RCT	19	35–79	80	G1: FLD G2: G-aenial	Anterior and posterior	Class V (NCCLs)	6	FDI	SAFR exhibited unacceptable performance
Sabbagh et al. (2017) [23]	Lebanon	RCT	34	6–12	68	G1: VF G2: PF	Molar	Class I	24	USPHS	SAFR performed similarly to CC
AlHumaid, Harbi, and ElEmbaby (2018) [24]	Saudi Arabia	RCT	20	*	40	G1: FLD G2: TNF	Anterior	Class V (CCLs)	18	USPHS	SAFR exhibited comparable performance to CC
Shaalan, Abou-Auf and Zoghby (2018) [25]	Egypt	RCT	18	19–40	18	G1: VF G2: z350	Molar	Class I	6	USPHS	SAFR exhibited similar performance to CC
Serin et al. (2019) [26]	Egypt	RCT	31	4–9	62	G1: VF G2: z250	Molar deciduous	Class I	12	USPHS	SAFRs predominantly showed alpha scores
Oz et al. (2020) [27]	Egypt	RCT	15	20	65	G1: VF G2: LF	Molar	Class I	60	FDI	SAFR demonstrated performance similar to CC
Maj et al. (2020) [14]	Poland	RCT	37	34–54	64	G1: VF G2: PF + OAO G3: PF + AE + OSP	Molar and incisor	Class I	24	USHPS	SAFR exhibited weaker results in terms of marginal adaptation and polishing.
Shaalan and Abou-Auf (2021) [28]	Egypt	RCT	18	19–40	36	G1: VF G2: z350 Xt	Molar	Class I	24	USHPS	SAFR showed performance comparable to CC
Kalola et al. (2022) [8]	India	RCT	30	18–54	60	G1: FLD G2: TNF	Molar	Class I	12	USPHS	SAFR exhibited satisfactory clinical performance.

RCT: Randomized Clinical Ttrial; G1: Group 1; G2: Group 2; G3: Group 3; VF: Vertise Flow (self-adhesive flowable resin, Kerr); FLD: Fusion Liquid Dentin (self-adhesive flowable resin, Pentron Clinical Technologies); TNF: Tetric N-Flow (conventional flowable resin, Ivoclar Vivadent); LF: LuxaFlow (conventional flowable resin, DMG); PF: Premise Flowable (conventional flowable resin, Kerr); 350: Filtek Z350 xt (conventional resin composite, 3M ESPE); 250: Filtek Z250 (conventional resin composite, 3M ESPE); NCCLs: Non-carious Cervical Lesions; CCLs: Cervical Carious Lesions; * Not mentioned; OAO: OptiBond All-In-One (all-in-one adhesive, Kerr); AE: Acid Etching (phosphoric acid etching step); OSP: OptiBond Solo Plus (two-step etch-and-rinse adhesive, Kerr); SAFRs: Self-Adhesive Flowable Resin; CC: Conventional Flowable Resin. Part 2

**Table 4:** USPHS and FDI data extracted from included studies.

Author, Year	Cavity	Conventional Resin Composite		Self-Adhering Resin Flowable	
		Criteria	Failure/Total	Criteria	Failure/ Total
Çelik, Aka and Yilmaz (2015) [22]	Class V	Marginal adaptation	0/40	Marginal adaptation	0/13
		Retention	0/40	Retention	27/40
		Post-operative sensibility	0/40	Post-operative sensibility	0/13
		Marginal staining	0/40	Marginal staining	0/13
		Color Match	0/40	Color Match	0/13
		Secondary caries	0/40	Secondary caries	0/13
		Anatomical Form	0/40	Anatomical Form	0/13
Sabbagh et al. (2017) [23]	Class I	*		*	
AlHumaid, Harbi, and ElEmbaby (2018) [24]	Class V	Marginal adaptation	3/20	Marginal adaptation	0/20
		Marginal staining	1/20	Marginal staining	0/20
		Color match	2/20	Color match	0/20
		Surface roughness	1/20	Surface roughness	0/20
Shaalan, Abou-Auf and Zoghby (2018) [25]	Class I	Marginal adaptation	0/18	Marginal adaptation	0/18
		Retention	0/18	Retention	0/18
		Post-operative sensibility	0/18	Post-operative sensibility	0/18
		Marginal Staining	0/18	Marginal Staining	0/18
		Color Match	0/18	Color Match	0/18
Serin et al. (2019) [26]	Class I	Retention	0/29	Retention	0/29
		Post-operative sensibility	0/29	Post-operative sensibility	0/29
		Marginal Staining	0/29	Marginal Staining	0/29
		Anatomical form	0/29	Anatomical form	0/29
Maj et al. (2020) [14]	Class I	Retention	0/22	Retention	1/22
		Marginal Staining	0/22	Marginal Staining	0/22
		Color Match	0/22	Color Match	1/22
		Proper Smoothness	0/22	Proper Smoothness	0/22
		Anatomical form	0/22	Anatomical form	0/22
Oz et al. (2020) [27]	Class I	Marginal adaptation	0/24	Marginal adaptation	0/22
		Retention	1/24	Retention	0/22
		Post-operative sensibility	0/24	Post-operative sensibility	0/22
		Marginal staining	0/24	Marginal staining	0/22
		Color Match	0/24	Color Match	0/22
		Proper Smoothness	0/24	Proper Smoothness	0/22
		Secondary caries	0/24	Secondary caries	0/22
Shaalan e Abou-Auf (2021) [28]	Class I	Marginal adaptation	0/18	Marginal adaptation	0/18
		Marginal staining	0/18	Marginal staining	0/18
		Color Match	0/18	Color Match	0/18
		Proper Smoothness	0/18	Proper Smoothness	0/18
		Secondary caries	0/18	Secondary caries	0/18
		Anatomical Form	0/18	Anatomical Form	0/18
Kalola et al. (2022) [8]	Class I	Retention	0/30	Retention	0/30
		Post-operative sensibility	0/30	Post-operative sensibility	2/30
		Marginal staining	0/30	Marginal staining	0/30
		Color Match	0/30	Color Match	0/30
		Surface roughness	0/30	Surface roughness	0/30
		Secondary caries	0/30	Secondary caries	0/30
		Anatomical Form	0/30	Anatomical Form	0/30

* Not mentioned Charlie Score; Color codes: blue—Cass v; purple—Class I; green—Alpha score (successful); red—Charlie or FDI scores 4–5 (failure).

**Table 5:** GRADE approach.

Certainty Assessment							No. of Patients		Effect		Certainty
No of Studies	Study Design	Risk of Bias	Inconsistency	Indirectness	Imprecision	Other Considerations	SAFRs	CFR	Relative (95% CI)	Absolute (95% CI)	
Marginal Adaptation (follow-up: range 6 months to 5 years)											
5	RCT	NS ^a^	NS ^b^	NS ^c^	NS ^d^	VSA ^e^	0/110 (0.0%)	0/112 (0.0%)	RR 0.00 (−0.04 to 0.04)	-- per 1.000 (from 0 fewer to 0 fewer)	⊕⊕⊕⊕ High
Retention (follow-up: range 6 months to 5 years)											
3	RCT	NS ^e^	NS ^b^	NS ^c^	NS ^d^	VSA ^e^	0/70 (0.0%)	1/72 (1.4%)	RR −0.01 (−0.06 to 0.04)	14 fewer per 1.000 (from 15 fewer to 13 fewer)	⊕⊕⊕⊕ High
Post-operative sensibility (follow-up: range 6 months to 5 years)											
3	RCT	NS ^e^	NS ^b^	NS ^c^	NS ^d^	VSA ^e^	0/70 (0.0%)	0/72 (0.0%)	RR 0.00 (−0.04 to 0.04)	-- per 1.000 (from 0 fewer to 0 fewer)	⊕⊕⊕⊕ High
Color match (follow-up: range 1 year to 5 years)											
5	RCT	NS ^a^	NS ^b^	NS ^c^	NS ^d^	VSA ^e^	1/110 (0.9%)	0/112 (0.0%)	RR 0.00 (−0.03 to 0.04)	-- per 1.000 (from 0 fewer to 0 fewer)	⊕⊕⊕⊕ High
Marginal discoloration (follow-up: range 6 months to 5 years)											
5	RCT	NS	NS	NS	NS	VSA ^e^	0/110 (0.0%)	0/112 (0.0%)	RR 0.00 (−0.04 to 0.04)	-- per 1.000 (from 0 fewer to 0 fewer)	⊕⊕⊕⊕ High
Anatomic Form (follow-up: range 1 year to 2 years)											
3	RCT	NS	NS	NS	NS	VSA ^e^	0/70 (0.0%)	0/70 (0.0%)	RR 0.00 (−0.05 to 0.05)	-- per 1.000 (from 0 fewer to 0 fewer)	⊕⊕⊕⊕ High
Secondary caries (follow-up: range 1 year to 2 years)											
3	RCT	NS	NS	NS	NS	VSA ^e^	0/70 (0.0%)	0/72 (0.0%)	RR 0.00 (−0.04 to 0.04)	-- per 1.000 (from 0 fewer to 0 fewer)	⊕⊕⊕⊕ High
Smoothness (follow-up: range 1 year to 2 years)											
3	RCT	NS	NS	NS	NS	VSA ^e^	0/62 (0.0%)	0/63 (0.0%)	RR 0.00 (−0.05 to 0.05)	-- per 1.000 (from 0 fewer to 0 fewer)	⊕⊕⊕⊕ High

Legend: CI—confidence interval; RR—risk ratio; RCT—randomized clinical trials; NS—Not serious; VSA—Very strong association; SAFRs—Self Adhesive Flowable Resin; CFR—conventional flowable resin. Explanations: ^a^. All included studies presented low risk of bias, and just two studies had some concerns for incomplete data on the allocation method, incomplete data on blinding of participants and staff, and incomplete data about loss of follow-up; ^b^. All included studies presented heterogeneity under <50%; ^c^. Indirectness judge based on population, intervention, comparison, and outcome across studies; ^d^. All included studies presented low CI; ^e^. All studies presented RR < 0.02.

**Table 6:** Summary of Findings—SAFRs vs. CFRs in Posterior Restorations.

Outcome	RR	95% CI	n (SAFR/CFR)	*p*-Value	GRADE Certainty
Marginal Adaptation	0.00	−0.04 to 0.04	110/112	0.53	High
Retention	−0.01	−0.06 to 0.04	70/72	0.81	High
Post-operative Sensitivity	0.00	−0.04 to 0.04	70/72	0.62	High
Color Match	0.00	−0.03 to 0.04	110/112	>0.05	High
Marginal Discoloration	0.00	−0.04 to 0.04	110/112	>0.05	High
Anatomic Form	0.00	−0.05 to 0.05	70/70	>0.05	High
Secondary Caries	0.00	−0.04 to 0.04	70/72	>0.05	High
Smoothness	0.00	−0.05 to 0.05	62/63	>0.05	High

RR: Risk Ratio; CI: Confidence Interval; SAFRs: Self-Adhesive Flowable Resins; CFR: Conventional Flowable Resins.

## Data Availability

All the data are available within the manuscript.

## Supplementary Materials

The following supporting information can be downloaded at: https://www.mdpi.com/article/10.3390/jcm14165862/s1, Table S1: PRISMA 2020 Checklist [38]; Table S2: Search strategy.
